# Assessment of genetic diversity and structure of Bambara groundnut [*Vigna subterranea* (L.) verdc.] landraces in South Africa

**DOI:** 10.1038/s41598-021-86977-7

**Published:** 2021-04-01

**Authors:** Adré Minnaar-Ontong, Abe S. Gerrano, Maryke T. Labuschagne

**Affiliations:** 1 Department of Plant Sciences, University of the Free State, P.O. Box 339, Bloemfontein, 9300 South Africa; 2 Agricultural Research Council-Vegetable and Ornamental Plant Institute, Private Bag X293, Pretoria, 0001 South Africa

**Keywords:** Genetics, Plant sciences

## Abstract

With its drought tolerant and protein-rich properties, Bambara groundnut [*Vigna subterranea* (L.) Verdc.], an indigenous African legume crop can contribute immensely to food security. This miracle crop is used as food and for the enhancement of soil fertility in South Africa. Knowledge on the genetic diversity and structure among the Bambara groundnut landraces can pave the way for the effective use and cultivation of this crop in southern Africa, especially South Africa. The aim of this study was to assess the genetic diversity and structure among Bambara groundnut landraces collected across South Africa and compared to a limited number of accessions from southern Africa using SSR markers. Seventy-eight Bambara groundnut accessions were genotyped using 19 Bambara specific SSR markers. SSR loci explored in this study, were all polymorphic. A total of 127 alleles were detected with a mean of 6.7 alleles per locus. Allele diversity and frequency among genotypes varied from 0.21 to 0.85 with an average of 0.62 per locus. Genetic variation as described by the analysis of molecular variance indicated higher genetic diversity (92%) within landraces than between (8%) different landraces. Population structure analysis showed that three subpopulations existed, and most of the South African accessions were restricted to one subpopulation, indicating that Bambara landraces has the ability to form unique haplotypes in different environments. Information harnessed in this study is helpful for further use in breeding programs for crop improvement.

## Introduction

With an ever changing climate and increasing drought reports across the world, agriculture is suffering, as current crops struggle to adapt in the new conditions, impacting food security. Several African countries’ economies based on agriculture, struggle to keep afloat as staple crops battle to adapt, risking production and food supply^1–3^. Bambara groundnut [*Vigna subterranea* (L.) Verdc.], an indigenous legume crop also known as a poor man’s crop, is cultivated at low levels across the African continent, especially Sub-Saharan Africa. This protein-rich (18–26%), drought tolerant, under-utilized legume species has several medicinal benefits and is rated as the third most produced and consumed crop after groundnuts and cowpea in semi-arid Africa^4–6^, with Nigeria as the largest producer in Africa^3,7,8^. In some African countries, like Zimbabwe, Bambara is preferred to maize, groundnuts and cowpea^9^. Most of the Bambara groundnuts produced, is maintained as local populations and grown as landraces^3^. These landraces are preserved and maintained in gene/germplasm banks for their agricultural significance^10^. Nigeria has the highest number of accessions with Zambia and Zimbabwe ranking 2nd and 3rd. South Africa has the lowest number of accessions as the introduction of this crop has been very recent^3,11^.

In South Africa, the crop is used as food as well as for the enhancement of soil fertility due its ability to fix nitrogen in the soil, with production limited to Mpumalanga and KwaZulu-Natal^6,11^. The growth in South African production areas for this crop are restricted by various limitations which include good stable varieties. The potential of this ‘miracle’ crop has not yet been explored to its full extent in South Africa, while Shegro et al.^6^ suggested that information on genetic variability among available local accessions (landraces) of Bambara groundnut can contribute to an improvement program in this country. Screening these Bambara landraces and the assessment of the genetic diversity of this poorly understood minor legume can provide breeders with genetic resources to assist with crop improvement including, yield, biotic and abiotic stress tolerance as well as the adaptability of the crop to various environments, which can contribute to the enhancement and maintenance of food security^12^.

Research on the genetic diversity of Bambara groundnut landraces mostly rely on phenotypic descriptors^5,6,13^ with a few reports on the use of molecular resources using isozyme markers^14^, random amplified polymorphic DNA (RAPD) markers^15^, amplified fragment length polymorphism (AFLP) markers^16,17^, and diversity arrays technique (DArT) markers^5^. Molecular markers have significant potential to explore genetic diversity as they are stable and detectable in all plant tissues and not affected by environmental effects^18^. SSR markers are described as such molecular markers and can be used to enhance the improvement of the ability and precision of classical plant breeding. SSR markers have been successfully employed to effectively reveal and interpret the genetic diversity found between Bambara groundnut landraces^19–23^.

The genetic structure of any crop reflects the evolutionary history and the evolutionary potential of such a crop. Understanding of the genetic structure of a crop population assists in the evaluation and improvement of such a crop^5,24,25^. Somta et al.^23^ suggested that the exploitation of the genetic diversity of Bambara groundnut landraces, will increase its potential as food, feed and cultivation in diverse environments. Bambara groundnut landraces from major growing centres, including southern, western, eastern and central Africa, as well as Asian regions have been characterized to determine the level of genetic diversity within and between landrace germplasm^5,12,20–23^.

The aim of this study was to assess the genetic diversity and structure among Bambara groundnut landraces collected across South Africa and other regions in southern Africa using SSR markers for the cultivation and improvement of Bambara groundnut.

## Materials and methods

### Plant material

Seventy-eight Bambara groundnut accessions (Table 1) from South Africa (45), Botswana (9), Namibia (7), Swaziland (5), Zimbabwe (2), Malawi (1), Madagascar (1), Singapore (1) and of unknown origin (7) were genotyped using 19 Bambara specific SSR markers (Table 2)^19,21,22^.

**Table 1:** List of Bambara groundnut accessions used for genetic diversity analysis.

Genetic diversity code	Accession name	Origin	Seed colour
SA_13	73223 Marabastad	South Africa	Light brown
SA_14	76467 Gravelotte	South Africa	Light brown
SA_15	ETL 76469	South Africa	Light brown
SA_36	MV 40-38	South Africa	Cream white
SA_38	MV 51-5-1C	South Africa	Light brown
SA_37	MV 67-1	South Africa	Light brown
SA_39	MV 74-2	South Africa	Cream white
SA_41	MV 8817	South Africa	Cream white with black blotch
SA_12	PGR3 S1	South Africa	Cream white
SA_16	PGR3 S2	South Africa	Cream white
SA_17	PGR3 S3	South Africa	Cream white
SA_43	RF-6135	South Africa	Brown
SA_44	RF-6158	South Africa	Cream white
SA_47	RF-6180	South Africa	Dark brown
SA_48	RF-6188	South Africa	Dark brown
SA_49	RF-6221	South Africa	Light brown with black speckles
SA_50	RF-6234	South Africa	Dark brown
SA_53	RF-6274	South Africa	Light brown
SA_54	RF-6303A	South Africa	Cream white with dark blotches
SA_55	RF-6304	South Africa	Dark brown
SA_28	SB 10-1F	South Africa	Light brown
SA_7	SB 10-2A	South Africa	Dark brown
SA_11	SB 1-1	South Africa	Dark brown with speckles
SA_29	SB 11-1C	South Africa	Light brown with black speckles
SA_8	SB 12-3	South Africa	Light brown with black speckles
SA_34	SB 14-7B	South Africa	Cream white
SA_3	SB 16-5A	South Africa	Brown with black speckles
SA_4	SB 19-3A	South Africa	Dark brown
SA_5	SB 20-2A	South Africa	Cream white
SA_2	SB 2-1	South Africa	Cream white
SA_1	SB 4-1	South Africa	Dark brown
SA_31	SB 4-2B	South Africa	Cream white
SA_42	SB 4-2BB	South Africa	Dark brown
SA_6	SB 4-4A	South Africa	Light brown
SA_30	SB 4-4G	South Africa	Cream white
SA_32	SB 4-4H	South Africa	Cream white with redish blotch
SA_10	SB 8-3	South Africa	Light brown
SA_35	SB 8-3C	South Africa	Light brown
SA_18	Sel from V4 S3 Dalby	South Africa	Light brown
SA_20	WS 42 (AS)	South Africa	Dark brown
SA_22	WS 49	South Africa	Cream white with dark blotch
SA_23	ZB S1	South Africa	Dark brown
SA_24	ZB S2	South Africa	Dark brown
SA_26	ZR S3	South Africa	Light brown
BOT_1	AS 5	Botswana	Dark brown
BOT_2	AS 8	Botswana	Cream white with dark blotch
BOT_3	AS 9	Botswana	Cream white with dark blotch
BOT_4	AS 10	Botswana	Dark brown
BOT_5	AS 11	Botswana	Cream white
BOT_6	AS 12	Botswana	Light brown with speckles
BOT_7	AS 13	Botswana	Cream white
BOT_9	AS 17	Botswana	Cream white with black speckles
BOT_10	AS 18	Botswana	Cream white with dark blotch
MOS_1	MAD 3	Madagascar	Cream white
MAL_4	M5	Malawi	Black
NAM_7	07K1	Namibia	Dark brown with dark speckles
NAM_8	07K3	Namibia	Dark brown with dark speckles
NAM_6	Caprivi Sel 1	Namibia	Cream white
NAM_1	K 1	Namibia	Cream white with dark blotch
NAM_3	K 5	Namibia	Black
NAM_4	K 6	Namibia	Light brown
NAM_2	K3	Namibia	Light brown
SIN_1	Ex Singapore	Singapore	Light brown
SWAZ_2	S 1	Swaziland	Light brown
SWAZ_4	Sel2 from SWAZI V4 S1	Swaziland	Light brown
SWAZ_5	SWAZI V4	Swaziland	Dark brown
SWAZ_6	SWAZI V5	Swaziland	Dark brown
SWAZ_7	SWAZI V5A	Swaziland	Light brown
ZIM_1	Red Ex Zimbabwe	Zimbabwe	Dark brown
ZIM_2	ZIM 003	Zimbabwe	Cream white
ND_10	6050B	Not determined	Dark brown
ND_5	AB 16-5C	Not determined	Light brown with black speckles
ND_2	CAP S1	Not determined	Cream white
ND_4	S1 Sel2	Not determined	Black
ND_7	Score 1	Not determined	Dark brown
ND_8	Score 2	Not determined	Dark brown
ND_1	Sel ZEDRES 1	Not determined	Cream white
ND_3	Sel1 from ZR S4	Not determined	Dark brown

**Table 2:** Primer Sequence information and annealing temperature for the 19 Bambara specific SSR markers.

Marker	Forward	Reverse	Tm
G180B2-D11	GAGGAAATAACCAAACAAACC	CTTACGCTCATTTTAACCAGACCT	59.0
G196AB4-D5	CCACGTTCTGGTTGTGAGTAGATA	GTGCTTTCAGACCATTACTTGCTT	49.4
G240-7-B2-D12	TTTTGTTGTTGTATGAATCCAGTG	CCTCATCAGACGCTCATCATT	59.0
G240-9-B2-D14	GAACGAAGCCAGGATAATGATAGT	CGAAAGCGACAACTCACTACTAAA	59.0
G33AB4-D1	TGCTTCTTCAAGGAGGAAGTAAGT	ACAAACATACGCACAACAGAGAAT	59.0
G358B2-D15	TGACGGAGGCTTAATAGATTTTTC	GACTAGACACTTCAACAGCCAATG	59.0
mBam2co80	GAGTCCAATAACTGCTCCCGTTTG	ACGGCAAGCCCTAACTCTTCATTT	59.0
PR-7	GTAGGCCCAACACCACAGTT	GGAGGTTGATCGATGGAAAA	55.3
PR-15	AGGAGCAGAAGCTGAAGCAG	CCAATGCTTTTGAACCAACA	55.3
PR-16	CCGGAACAGAAAACAACAAC	CGTCGATGACAAAGAGCTTG	57.6
PR-18	TCTGCCACATTTCGCATAAG	CGCTTCAAATCCGATGTTCT	55.3
PR-19	AGGCAAAAACGTTTCAGTTC	TTCATGAAGGTTGAGTTTGTCA	55.3
PR-26	CGCTCATTTTAACCAGACCTC	CAAACAAACCAACGGAATGA	57.6
PR-30	AATGCAAGATTTTGGCTTGG	CCCACTCAAACCATACACCA	59.0
PR-32	TTCACCTGAACCCCTTAACC	AGGCTTCACTCACGGGTATG	57.6
PR-33	ACGCTTCTTCCCTCATCAGA	TATGAATCCAGTGCGTGTGA	57.6
PR-37	CCGATGGACGGGTAGATATG	GCAACCCTCTTTTTCTGCAC	55.3
PR-44	TGTGGGCGAAAATACACAAA	TCGTCGAATACCTGACTCATTG	59.7
PR-48	TACCTGCATTCGGGACAGTT	TTCACTCTTTCTTGATCACATGC	59.0

### DNA isolation

Total genomic DNA of each accession was extracted from young leaves using the GenElute Plant Genomic DNA kit (Sigma Aldrich) according to manufacturer’s instructions^20^. DNA quantity and quality were estimated from a 0.8% (w/v) agarose gel with electrophoresis at 80 V in UNTAN (40 mM Tris–Cl; 2 mM EDTA, pH adjusted to pH 7.4 with acetic acid) buffer. DNA was visualized with ethidium bromide staining under UV light. The concentration of the DNA samples was determined by using a UV spectrophotometer and measuring absorbance at A_260_ and A_280_. Samples were diluted to 20 ng/μl for SSR analysis.

### SSR analysis

Each PCR amplification reaction contained 40 ng DNA, 1 × KAPATaq ReadyMix DNA polymerase, 50 ng each of the forward and reverse primer (Integrated DNA technologies) and 0.1 mg/ml Bovine serum albumin (BSA) in a total reaction volume of 10 μl. The optimized cycling conditions for the primers used were: 94 °C for 2 min, 35 cycles of 94 °C for 1 min, 45–60 °C (depending on the primer) for 1 min and 72 °C for 30 s followed by a final extension of 72 °C for 5 min and a 10 °C hold. Polymerase chain reaction products for all markers were separated on a 5% (w/v) non-denaturing polyacrylamide gel using the GelScan 3000 Real-Time DNA Fragment Analysis system with software version 8.00.01 (Corbett Research, Sydney, Australia). The alleles were scored as present (1) or absent (0) based on the size of the amplified product using a 25 bp DNA ladder (Promega, Madison, WI, USA).

### Data analysis

Scored data were used to construct a binary data matrix for statistical analysis. Different statistical programs were used for genetic diversity analyses. Total allele number (Na) and average number of alleles at each loci were calculated manually. Allelic polymorphic information content (PIC) was calculated from the binary data of the 19 SSR markers using iMEC: Online Marker Efficiency Calculator developed by Amiryousefi et al.^26^. PIC evaluates polymorphism of a marker by characterizing the efficiency of each primer for detecting polymorphic loci^27^. A PIC of > 0.5 indicates high diversity, a PIC < 0.25 low diversity and a PIC between 0.25 and 0.5 intermediate diversity^28^. As part of the assessment of the genetic diversity among the Bambara groundnut landraces an analysis of molecular variance (AMOVA) was done using GenAlex 6.51b2^29,30^. A rooted, unweighted neighbor-joining (NJ) dendrogram with 30,000 bootstrap repetitions was constructed with DARwin 6.0.19^31^. Genetic similarities between landraces were compared by using the Jaccard similarity index^32^. Cluster analysis information is sensitive to closely related individuals, therefore the constructed dendrogram illustrates the relationship among the Bambara groundnut landraces based on SSR allele variation. The Bayesian clustering method implemented in the STRUCTURE 2.3.4 software^33^ was used to infer clusters of the landraces. Analysis from the STRUCTURE software was used to estimate the ‘true’ number of populations (*K*) without prior knowledge of the population^34,35^. The K value was first calculated using a burn-in length of 10^4^ and 10^4^ Monte Carlo Markov Chain (MCMC) repetitions using 10 iterations for K1 to K10 using optimum K from CLUMPAK^36^. The dataset was then re-analyzed using the best calculated K value with a burn-in length of 10^5^ and 10^5^ MCMC replicates to determine the final population groupings.

## Results

### SSR marker analysis

The 19 SSR loci explored in this study, were all polymorphic and produced varying number of alleles with different size ranges (Table 3). A total of 127 alleles were detected with a mean of 6.7 alleles. The number of alleles per locus ranged from three for SSR marker PR-44 to eleven for G240-9-B2-D14. The mean alleles observed was higher than the 5.20 reported by Basu et al.^19^ who also used some of the SSR markers employed in this study. Polymorphic information content (PIC) value is an indication of allele diversity and frequency among genotypes. The PIC values varied significantly among the different markers used in this study. The values ranged from 0.21 for marker PR-7 to 0.85 for marker mBam2co80 (Table 3) with an average of 0.62 per locus. High PIC estimates reflect the strength of the DNA markers, especially SSRs as co-dominant markers.

**Table 3:** SSR marker, allele number, sizes and polymorphic information content.

Marker	Number of alleles	Allele size range (bp)	PIC value
G180B2-D11	6	170–390	0.47
G196AB4-D5	6	205–390	0.64
G240-7-B2-D12	7	140–225	0.73
G240-9-B2-D14	11	122–225	0.84
G33AB4-D1	6	140–270	0.74
G358B2-D15	9	160–303	0.71
mBam2co80	6	207–250	0.85
PR-7	6	175–300	0.21
PR-15	5	187–390	0.55
PR-16	9	160–450	0.50
PR-18	5	199–225	0.60
PR-19	8	240–300	0.60
PR-26	7	150–225	0.73
PR-30	10	200–430	0.34
PR-32	7	175–400	0.63
PR-33	6	140–230	0.50
PR-37	6	200–300	0.64
PR-44	3	175–225	0.78
PR-48	4	180–250	0.69

### Analysis of genetic variance and structure of the Bambara groundnut landraces

#### Analysis of molecular variance

AMOVA was performed to test the genetic structure of the Bambara groundnut landraces. Based on the AMOVA results, most of the genetic variation (92%) (Table 4) was due to variation within landraces and only a small portion (8%) of the variation was due to genetic diversity between different landraces. The genetic variation within landraces is referred to the genetic variation within landraces from the same production area, while the variation between refers to the genetic variation compared between landraces from the different production areas.

**Table 4:** Genetic variation of Bambara groundnut landraces.

Source of variation	df	Sum of squares	Estimated variation	% variation	P-value
Between landraces	2	96.768	1.314	8.0	< 0.001
Within landraces	75	1131.296	15.084	92.0	< 0.001
Total	77	1228.064	16.398		

#### Rooted cluster analysis

**Figure 1:**
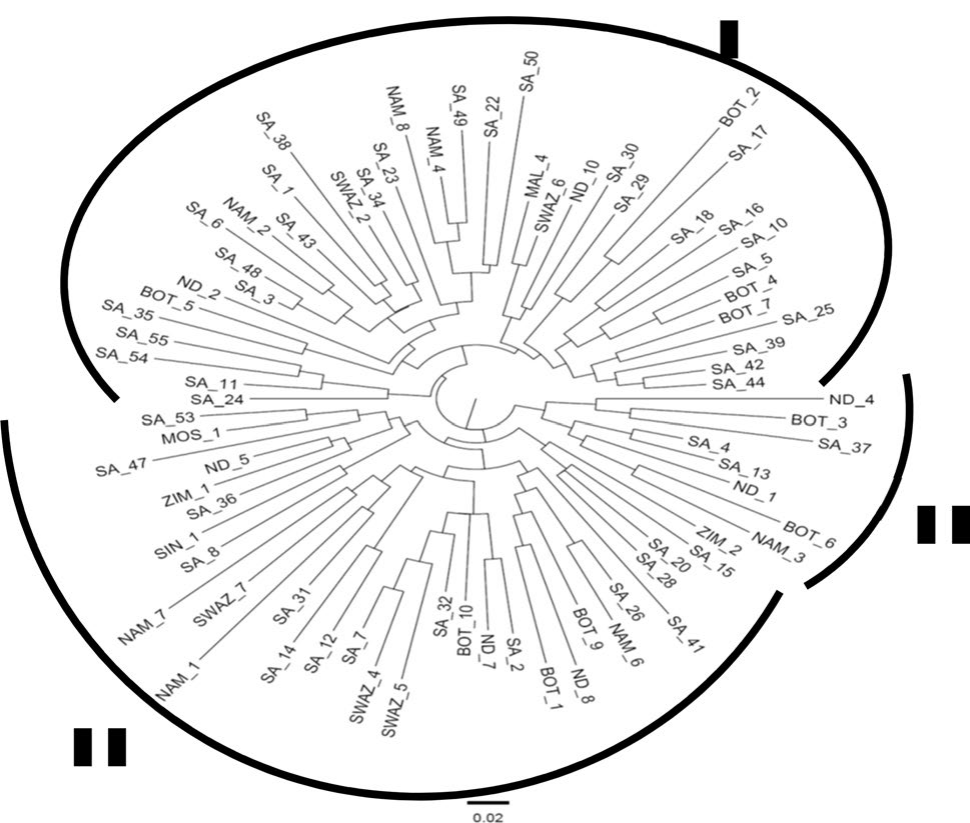
Rooted neighbor-joining (NJ) tree constructed with DARwin 6.0.19 software, illustrating clusters for the 78 Bambara groundnut landraces.

Three clear and distinct clusters were observed from the rooted cluster analysis from the different origins using DARwin 6.0.19 software (Fig. 1), but there was no relationship between SSR clusters and geographic origin. All three clusters were subdivided into 2 sub-clusters.

Cluster I, which was divided into two sub-clusters include landraces from South Africa (27), Botswana (4), Namibia (3), Swaziland (2), Malawi (1) and two landraces (ND_2 and ND_10) from unknown origin (ND). This cluster also contained the most South African and Botswana landraces and none of the Zimbabwean landraces. This cluster contain 39 landrace accessions of which the seed colour indicated to be predominantly dark brown (38%), followed by cream white (33%), light brown (26%) and only 3% black seeds. The landrace from unknown origin (ND_2) in sub-cluster 1 of cluster I showed no relation with any of the landraces from the known or unknown origins. The landrace from unknown origin (ND_10) however, showed 73% similarity with one of the SA landraces (SA_30). Even though these accessions share a high genetic similarity, they differ based on seed colour where the ND_10 accession have dark brown seeds and the SA_30 accession have cream white seeds. Genetic variation of 6% similarity between a SA landrace (SA_24) and a cluster of 3 other SA landraces (SA_11, SA_54 and SA_55) was observed in one of the sub-clusters of Cluster I. The seed colour of accessions in this sub-cluster, are predominantly dark brown with only one accession (SA_54) with cream white seeds with dark blotches.

Cluster II consisted of landraces from SA (15), Botswana (3), Namibia (3), Swaziland (3), Zimbabwe (2), Singapore (1), Madagascar (1) and three landraces from unknown origin (ND). This cluster was divided into two sub-clusters. This is the only cluster that included Zimbabwean landraces. More accessions from cluster II indicated to have cream white seeds (39%) followed by accessions with light brown (32%) and dark brown (29%) seeds. This cluster have no accessions with black seeds. Sub-cluster two from cluster II contained mostly SA landraces and one from Zimbabwe. Seed colour within this sub-cluster is observed to be mostly variations of brown, while only one accession had cream white seeds. The landraces from unknown origin (ND_7 and 8) in cluster II showed 90% and 82% similarity with a SA (SA_2) and Botswana (BOT_1) landrace, respectively. The high genetic similarity between ND_7 and SA_2 again shows that there is no correlation between seed colour and the genetic similarity of different landraces. The landrace from unknown origin (ND_5), showed 67% similarity with one of the landraces from Zimbabwe. Furthermore, the genetic variation between landraces varied between 87% similarity between a landrace from the South African germplasm collection (SA_7) and a landrace from Swaziland (SWAZ 4). In this case however, both accessions had dark brown seeds.

Cluster III is the cluster with the lowest number of landraces (8). These included landraces from SA, Botswana, Namibia and two landraces from unknown origin (ND). This small cluster was divided into two sub-clusters. Seed colour for this cluster indicated to be predominantly light brown (38%), followed by equal contributions to cream white (25%) and black (25%) and only 12% dark brown seeds. The landrace from unknown origin (ND_1) in sub-cluster 1 of cluster III showed significant genetic similarity as but differed in seed colour with landraces from Namibia and Botswana. This also describes the relationship of a landrace from unknown origin (ND_4) in sub-cluster 2, with landraces from SA and Botswana. No clones (100% similarity) were observed in any of the main clusters.

#### Bayesian model-based clustering

**Figure 2:**
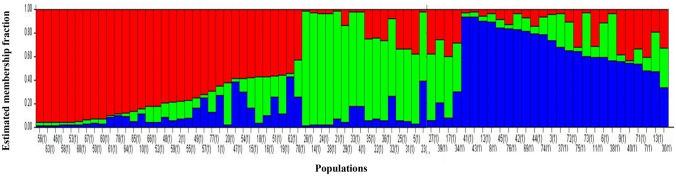
Estimated genetic structure at K = 3 for the 78 Bambara groundnut landraces using the admixture model of population structure. Population 1 is indicated in red, population 2 in green and population 3 in blue.

Structure analysis delineates clusters of individuals based on their genotype by using Bayesian model-based clustering. This analysis is done without prior knowledge of the genotypes. The optimum number of genetic clusters for the 78 landraces was determined at *K* = 3 as identified by Evanno et al.’s^34^ ad hoc* ΔK* method. The 78 landraces divided into three populations (*K* = 3) (Fig. 2), which is the minimum observed number of populations using this protocol. Thus all of the landraces could have been part of a single population. Structure analysis with three populations *K* = 3 (Fig. 2) identified three clusters—Population 1 (red) composed of landraces from SA, Botswana, Namibia, Swaziland and some of unknown origin. Population 2 (green) containing the bulk of the landraces from South Africa with a mixture of landraces from six of the other origins and one land race from the unknown origin, Population 3 (blue) contained most of the landraces from Botswana. These results confirmed the genotypic diversity observed within the landraces using cluster analysis.

Population 1 consisted mainly of the genetic contribution of Swaziland landraces (60%) and landraces of unknown origin (57%) (Table 5). A moderate percentage of the genetic contribution of landraces from Botswana, Namibia and South Africa was assigned to this population, ranging from 14% for Namibia to 22% for both SA and Botswana. Population 2 contained the highest fraction of South African landraces (53%), sharing equal fractions of Nambian (43%) and Zimbabwean (50%) landraces with population 3. The highest genetic contribution towards population 3 came from the Botswana landraces, with 67%.

**Table 5:** Genetic contribution of 78 Bambara groundnut landraces from the geographic origins in three populations as detected by structure analysis.

Geographic origin	Inferred clusters			Number of individuals
	Population 1	Population 2	Population 3	
Botswana	0.22	0.11	0.67	9
Madagascar	0.00	1.00	0.00	1
Malawi	0.00	1.00	0.00	1
Namibia	0.14	0.43	0.43	7
Singapore	0.00	0.00	1.00	1
South Africa	0.22	0.53	0.25	45
Swaziland	0.60	0.40	0.00	5
Zimbabwe	0.00	0.50	0.50	2
Unknown	0.57	0.14	0.29	7

## Discussion

All loci evaluated, was polymorphic. The use of SSR markers enables comparison of allelic diversity and PIC value. Only 11% of the 19 markers employed generated PIC values between 0.25 and 0.5 indicating moderate usefulness, while 84% of the markers employed generated PIC values higher than 0.5, indicating significantly high usefulness and is recommended to determine genetic diversity among Bambara groundnut landraces. The PIC value of each SSR marker can be evaluated based on the alleles detected by that specific marker, but the current PIC results indicate that the marker that yields the most alleles is not necessarily the most informative primer combination. The detection of unique alleles allocated to different regions of origin, was limited to South Africa as a very low number of accessions was analyzed for the other regions.

Most of genetic diversity investigations documented high levels of genetic diversity among Bambara groundnut landraces sampled across large geographic regions^12,21,22^. Limited studies of the genetic structure of Bambara landraces have been conducted^21,23^. In the current research, most of the genetic variation in Bambara groundnut landraces (92%) was found common to all of the populations, with a much lower percentage (8%) of genetic variation limited to one or more populations, suggesting significant gene flow among the landraces from the different origins, across large geographical distances.

Some of the earlier studies from outside South Africa found low genetic diversity within groups and a higher diversity among groups. Most of the literature agrees that Bambara groundnut landraces show high levels of genetic diversity among rather than within groups. This study is the first in South Africa and confirms previous studies^22,37^ where higher levels of genetic diversity within groups rather than among groups were reported. However, the genetic diversity observed for Bambara groundnut landraces depends on the techniques used and the number of individuals evaluated.

Bambara groundnuts are self-pollinators, described as predominantly homozygous^38^ and highly inbred, suggesting a limited amount of gene flow and therefore low levels of genetic diversity between groups. The adaptability of this self-pollinating crop to various environments promotes the genetic diversity observed within the groups. Three subpopulations were observed, where most of the South African accessions were restricted to one subpopulation. South Africa has the lowest accession number of Bambara landraces, and the production areas are very limited. This can explain the restriction of the landraces to one populations. This can also mean that the South African landraces possesses unique alleles and has the potential to be explored more intensely than what was done during this study.

The genetic structure of a population can be influence by several factors that range from gene flow (migration) and population size to the ability of the population to adapt to changes in the environment. These influential factors makes it difficult to correlate the genetic structure of a population to the geographical distribution of individuals involved^34,39^, as was observed for the 78 Bambara groundnut landraces used in this study. The observed clustering patterns are quite different from the results reported by previous researchers as the landraces included in this study did not cluster based on origin or seed colour. The statistical analysis methods used in this study all concluded that the three identified populations have more genetic diversity within than among the groups, because of the integration of landraces from the diverse origins.

## Conclusion

Understanding the genetic variability of Bambara landraces in South Africa will increase the efficiency of future crop improvement programs in the country and will encourage the expansion of Bambara production areas. Consequently, this could potentially contribute to increase farmer, especially small-scale producer’s income, better food security, especially in regions of water scarcity and improve the quality of local diets within the region. The genetic diversity of Bambara groundnut evaluated in this study is relatively higher within than among the landraces. This is confirmed by the self-pollinating characteristics of the crop, as well as the slow gene flow identified between the diverse origins. The seed colour recorded for the landraces, differ significantly and could not be correlated with the genetic clusters. Future recommendations would include the addition of more landraces and a more intense study on the morphological traits, because the knowledge of the genetic diversity and population structure of Bambara groundnut landraces can contribute to the cultivation and improvement of this crop.

## Data Availability

Data and material are available if needed.
